# Late postpancreatectomy hemorrhage from the gastroduodenal artery stump into an insufficient hepaticojejunostomy: a case report

**DOI:** 10.1186/s13256-021-02743-3

**Published:** 2021-04-29

**Authors:** Adam Zeyara, Bobby Tingstedt, Bodil Andersson

**Affiliations:** 1 Department of Clinical Sciences Lund, Surgery, Lund University, Lund, Sweden; 2 Department of Surgery, Ystad Hospital, Ystad, Sweden; 3 Section of Hepato-Pancreato-Biliary Surgery, Department of Surgery, Skåne University Hospital, SE-221 85 Lund, Sweden

**Keywords:** Pancreatoduodenectomy, Postpancreatectomy hemorrhage, Gastroduodenal artery, Morbidity, Case report

## Abstract

**Background:**

Mortality after elective pancreatic surgery in modern high-volume centers is very low. Morbidity remains high, affecting 20–40% of patients. Late postpancreatectomy hemorrhage is a rare but potentially lethal complication. The exceptionality in our case lies in the underlying mechanism of its clinical presentation. It is a demonstration of the difficulties associated with finding the source of bleeding in late postpancreatectomy hemorrhage.

**Case presentation:**

An 82-year-old White female was diagnosed with a periampullary malignancy and underwent pancreatoduodenectomy. Postoperatively, the patient suffered from an anastomotic leak in the hepaticojejunostomy, which was treated with percutaneous pigtail drains in the abdomen and in the biliary tract. On the fourth postoperative week she presented blood in both drains and in her stool. Given our knowledge about the biliary anastomotic leak, this presentation led us to suspect an intraluminal source (biliary tract or gastrojejunostomy) with blood leaking through the insufficient hepaticojejunostomy into the abdominal cavity. Upper tract endoscopy and computed tomography angiography were, however, unremarkable. Further investigation with conventional angiography identified the bleeding source at the gastroduodenal artery stump, which was successfully coiled. Hence, the gastroduodenal artery stump was bleeding into the insufficient hepaticojejunostomy, filling up the biliary tree and the small intestine. After coiling of the artery, the remainder of the postoperative care was uneventful.

**Conclusion:**

Postpancreatectomy hemorrhage presents a major clinical challenge after pancreatoduodenectomy, with significant morbidity and high risk for mortality. The treating physician must be alert and active in the investigation and treatment of the bleeding source to ensure a successful outcome.

## Background:

Mortality after elective pancreatic surgery in modern high-volume centers is less than 3% [1–3]. Morbidity, however, remains high, affecting 20–40% of patients [1]. The most frequent causes for morbidity are pancreatic fistulas, other anastomotic leaks, and delayed gastric emptying [1–4]. Postpancreatectomy hemorrhage (PPH) is a less frequent but potentially lethal complication, occurring in 3–13% of patients after elective pancreatic surgery [1, 3, 5–7]. According to the International Study Group of Pancreatic Surgery (ISGPS), PPH is either early (< 24 hours) or late (> 24 hours), extra- or intraluminal, and mild or severe with different clinical conditions; it can have diagnostic and therapeutic consequences and is graded into A-, B-, or C-type bleeds (Table 1) [5].

**Table 1:** Proposed definition of postpancreatectomy hemorrhage according to the International Study Group of Pancreatic Surgery

Grade	Time of onset, location, severity, and clinical impact		Clinical condition	Possible diagnostic consequences	Possible therapeutic consequences
A	Early intra- or extraluminal, mild		Well	Observation, blood count, radiology	No
B	Early, intra- or extraluminal, severe	Late, intra- or extraluminal, mild	Often well/intermediate, very rarely life threatening	Observation, blood count, CT, angiography, endoscopy	Transfusion of fluid/blood, intermediate or intensive care unit, endoscopy, coiling, relaparotomy if early PPH
C		Late intra- or extraluminal, severe	Severely impaired, life threatening	Angiography, CT, endoscopy	Angiography, coiling, endoscopy, relaparotomy, intensive care unit

Adapted from Wente *et al.* [5]

The main cause of early PPH is a physiologic or technical failure of hemostasis such as bleeding from an anastomosis, diffuse bleeding due to coagulopathy, or a slipped ligature or metal clip [1, 3]. Late PPH is often associated with the presence of intraabdominal erosive factors such as anastomotic leaks and/or intraabdominal infections [8]. Late hemorrhage carries a significant mortality rate of 15–58% [3].

The centralization of pancreatoduodenectomy (PD) has led to decreased PD-specific complications and better overall survival [2]. The implementation of enhanced recovery after surgery (ERAS)-type protocols has positively affected general postoperative morbidities such as delayed gastric emptying—however, PD-specific complications (including PPH) remained unaffected [4].

We present a case of late PPH with a rare mechanism of presentation due to other coexisting complications, demonstrating the complex nature of postoperative care in advanced pancreatic surgery.

## Case presentation

The patient is an 82-year-old White female with a history of high blood pressure, hyperlipidemia, hypothyroidism, and dyspepsia; she had previously undergone a cholecystectomy because of gallstones (50 years ago) and a hysterectomy because of cancer (40 years ago). Her medications included metoprolol (50 mg once daily), simvastatin (10 mg once daily), levothyroxine (150 μg once daily) and omeprazole (20 mg once daily). She was evaluated at our tertiary hepatopancreatobiliary center because of an asymptomatic double-duct sign. Further assessment with magnetic resonance imaging (MRI) and endoscopic retrograde cholangiopancreatography (ERCP) concluded a diagnosis of periampullary malignancy. Laboratory work was unremarkable, including bilirubin levels, international normalized ratio (INR), and albumin levels. After a multidisciplinary conference, the patient was accepted for pancreatoduodenectomy.

A pylorus-preserving pancreatoduodenectomy and lymphadenectomy was performed through a midline incision. The patient was reconstructed with a pancreatojejunostomy, hepaticojejunostomy, and gastrojejunostomy on a single jejunal loop. Two BLAKE (silicone) drains were introduced, one behind the hepaticojejunostomy and one behind the pancreatojejunostomy. According to the operation report, there were no significant perioperative events.

**Fig. 1:**
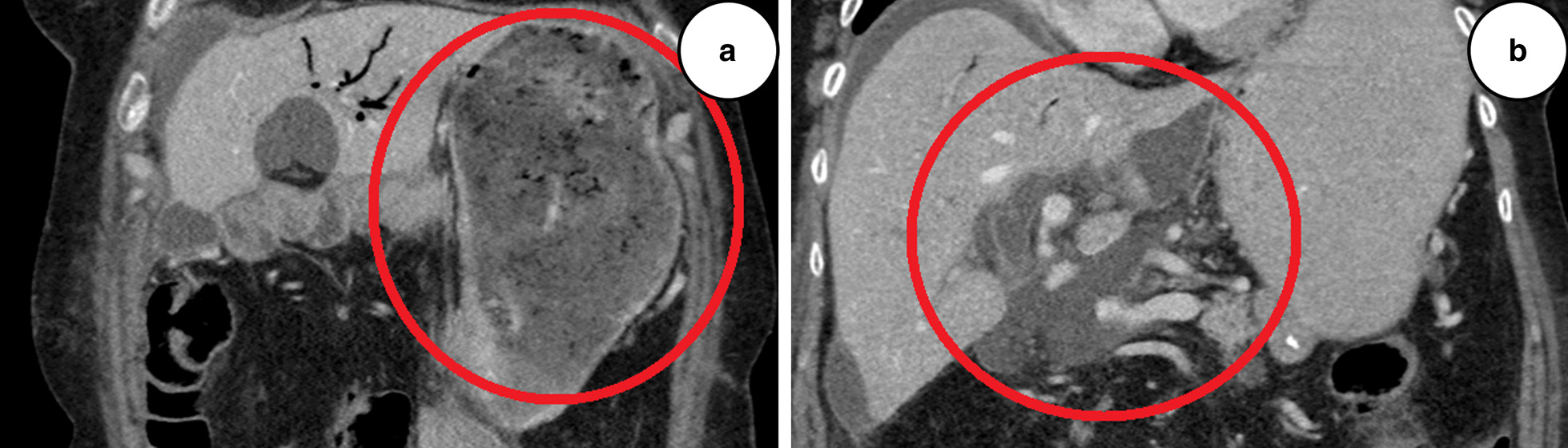
**a** Computed tomography, coronal section of the upper abdomen, postoperative day 10, showing gastric retention. **b** Computed tomography, coronal section of the upper abdomen, postoperative day 10, showing free low-density fluid around the liver and in the vicinity of hepaticojejunostomy, consistent with insufficient hepaticojejunostomy

Postoperative days (POD) 1–5 were uneventful. Both drains (peak amylase levels 20 and 60 U/L) were successfully removed. On POD 7, an increased C-reactive protein (CRP) of 270 mg/L was observed. Computed tomography (CT) of the lungs and abdomen revealed peribronchial infiltrates and a small pool of free fluid in the upper right quadrant of the abdomen. The clinical condition was unremarkable, and the patient was started on intravenous antibiotics, piperacillin and tazobactam. On POD 10, a repeat CT was performed showing a large gastric retention and findings consistent with insufficient hepaticojejunostomy (Fig. 1a, b). The gastric retention was treated with a nasogastric tube, and the patient was started on parenteral nutrition. An ultrasound-guided percutaneous intraabdominal pigtail drain was established. The drain discharge was bilious to the naked eye, and the bilirubin level was 320 μmol/L. Discharge levels reached 1000 mL/day, and on POD 12 a percutaneous transhepatic cholangiography (PTC)-guided 8-French (Fr) pigtail drain was advanced through the anastomosis to deviate the leak from the abdomen.

**Fig. 2:**
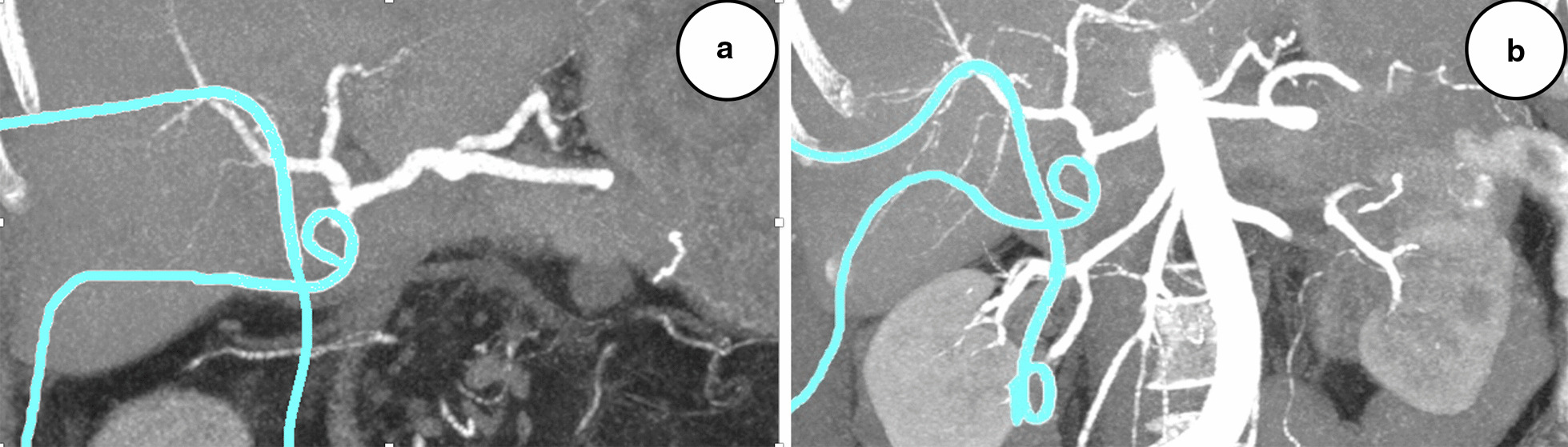
**a** Computed tomography angiography, coronal section of the upper abdomen, postoperative day 26, showing the celiac trunk and all main branches (splenic, left gastric, and common hepatic arteries), as well as the gastroduodenal artery stump, the proper hepatic, right hepatic, and left hepatic arteries, with no evidence of bleeding. Pigtail drains can be seen in turquoise. **b** Repeat computed tomography angiography of the upper abdomen performed on day 28, also without evidence of bleeding from any of the above-mentioned vessels. Pigtail drains can be seen in turquoise

**Fig. 3:**
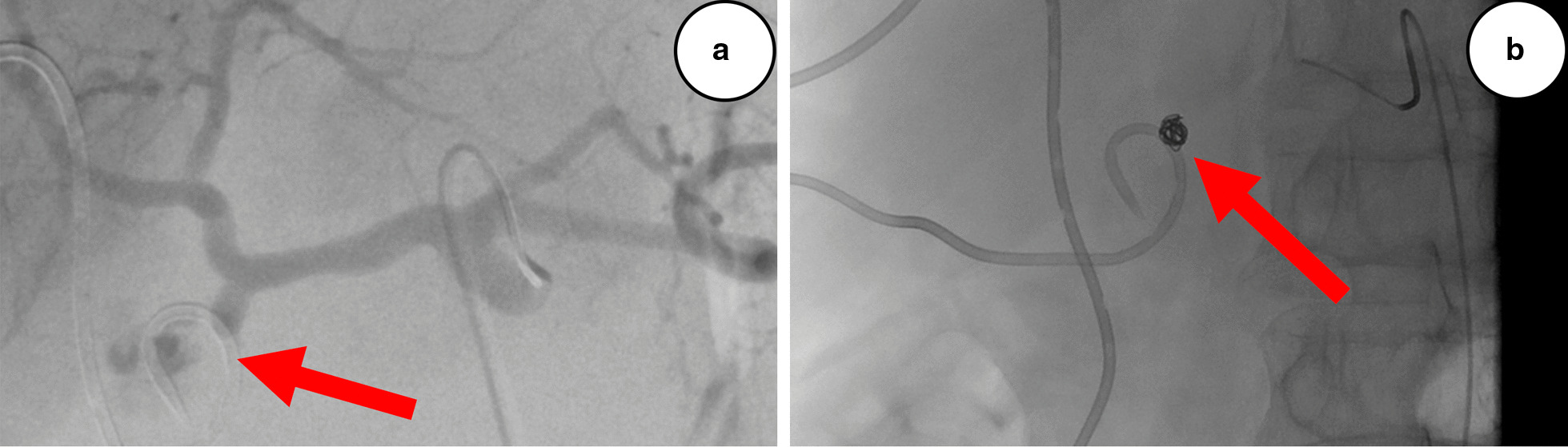
**a** Conventional selective angiography on postoperative day 29 showing contrast leakage (arrow) from the gastroduodenal artery stump. **b** Successfull coiling (arrow) with endovascular technique

**Fig. 4.**
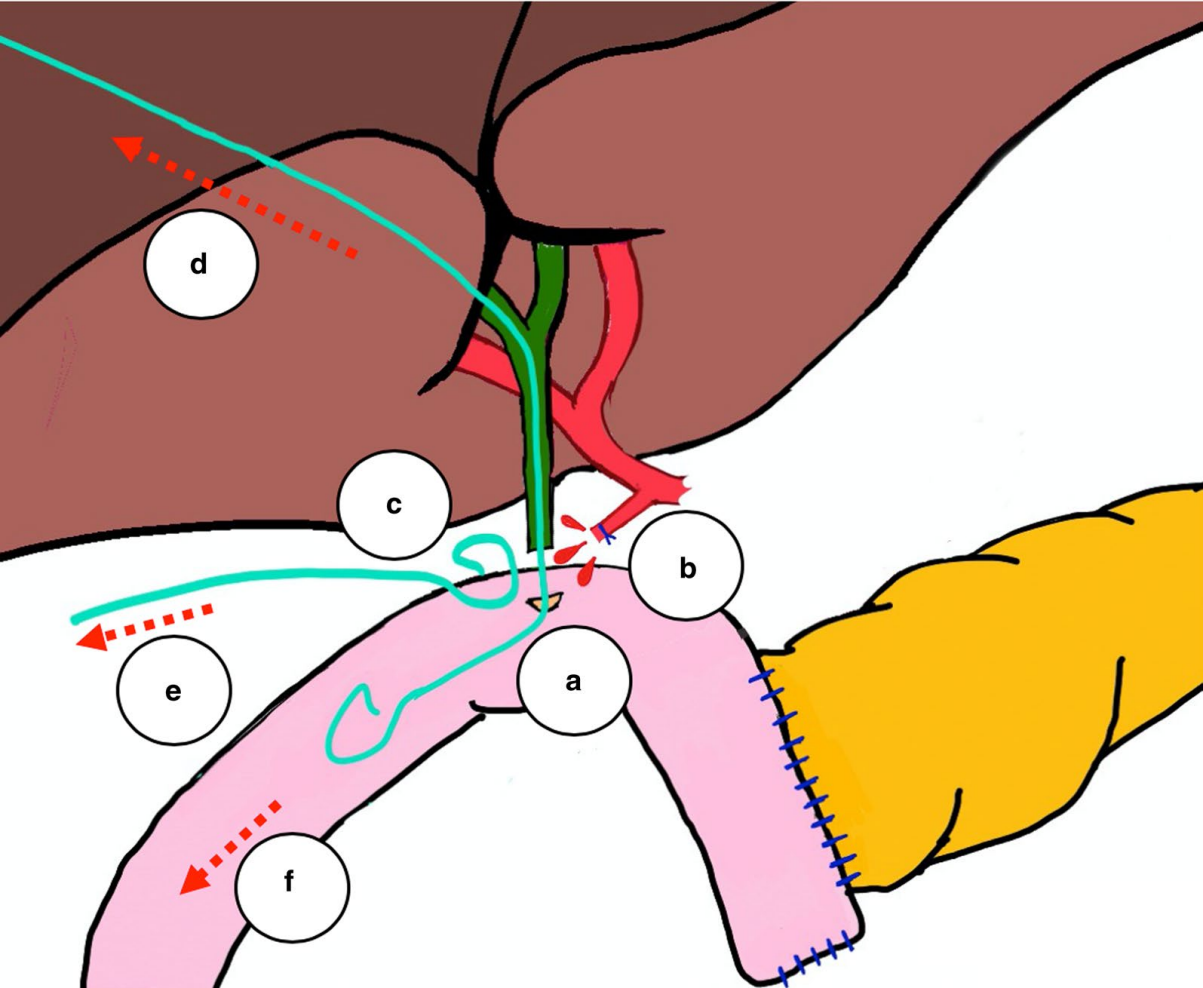
Schematic illustration of the altered anatomy after a modified pylorus-sparing pancreaticoduodenectomy and our rationale of the observed postoperative pathology. **a** Leaking hepaticojejunostomy with transhepatic pigtail drain. **b** Bleeding from the gastroduodenal artery stump. **c** Percutaneous intraabdominal pigtail drain. **d** Entering of blood into transhepatic pigtail drain via the insufficient hepaticojejunostomy, mimicking an intraluminal bleeding source. **e** Entering of blood into percutaneous intraabdominal pigtail drain, suggesting an extraluminal bleeding source. **f** Entering of blood into bowel through the insufficient hepaticojejunostomy, mimicking an intraluminal bleeding source

On POD 26, the patient presented blood in both drains and bloody stools. Endoscopy of the gastrojejunostomy (the hepaticojejunostomy could not be visualized because the reconstruction was made on a long Roux loop) and a CT angiography (Fig. 2a) could not localize any evidence of bleeding. The patient was hemodynamically stable, and there was only discrete evidence of bleeding on the hemoglobin curve, presently 89 g/L (98 g/L on the day before and had been stable at approximately 100 g/L without transfusions during the entire preceding postoperative period). Two units of erythrocytes were administered, and on the next day the hemoglobin level was 107 g/L. On POD 28, the patient had a new episode of bleeding in both drains and multiple bloody stools. A repeat endoscopy and CT angiography (Fig. 2b) were both unremarkable. Besides discrete pallor and tiredness, the patient was still in good clinical condition with stable vitals; hemoglobin level was 86 g/L, and after one erythrocyte transfusion it was 93 g/L. Because of continuous bleeding but no identified source, an empiric suspicion of intraabdominal bleeding was raised, and a conventional selective angiography was performed on the next day, revealing a contrast leakage from the gastroduodenal artery (GDA) stump (Fig. 3a), which was coiled successfully (Fig. 3b). The biliary drain had to be changed because it was completely obliterated by blood clots. The clinical conclusion was that of a grade B hemorrhage, and that the blood from the GDA stump had traveled through the insufficient hepaticojejunostomy into the biliary tree and small intestine, causing the presence of blood in both drains and bloody stools (Fig. 4). During the next couple of days, the intraabdominal drain was removed, and the patient was discharged with the biliary drain in situ on POD 45. Her hemoglobin level was 93 g/L, and inflammatory parameters and liver function tests were unremarkable.

On POD 75, she was evaluated in the outpatient clinic and the biliary drain could be removed after having been closed for 10 days.

The pathology report revealed an ampullary adenocarcinoma. Adjuvant chemotherapy was not advised because of low tumor stage (pT1bN0R0), highly differentiated intestinal histology, and the time elapsed since the primary surgery and the associated postoperative morbidity.

## Discussion

A combination of extra- and intraluminal presence of blood is not a spectacular finding in the presence of an insufficient hepaticojejunostomy and a transhepatic drain. Also, bleeding from the GDA stump is the most frequent origin of PPH [9], but it is the underlying mechanism in our case that is exceptional. The presentation initially led us to believe that the source of bleeding was intraluminal. Given our knowledge of the biliary anastomotic leak, the bloody discharge from the abdomen was assumed to have leaked into the peritoneal cavity through the insufficient hepaticojejunostomy. In fact, it was the other way around—a long stump of GDA adjacent to the anastomotic defect was bleeding into the biliary tree and small intestine.

The erosive milieu due to anastomotic leaks and its association with late PPH is uncontroversial. In this case, a diagnosis of biliary leakage was perhaps delayed by some 3 to 4 days, which theoretically could have affected the GDA stump negatively. Visceral artery pseudoaneurysms after pancreatoduodenectomy are rare but well-known phenomena. Gastroduodenal artery stump pseudoaneurysms are the most common, with an incidence of 0.2–8.3% [7]. Most of them have a preceding complication in the postoperative period [7]. To the authors’ knowledge, there are no large series examining the diagnostic yield and potential survival benefit of performing follow-up CT angiographies in selected high-risk cases after pancreatic surgery.

In a recent systematic review, CT angiography and conventional angiography had the same sensitivity (67 versus 69%) for identifying the origin of late PPH [9]. In our case, two consecutive CT angiographies were negative. Only when a catheterization of the celiac trunk was made could we identify the source of bleeding. It was confirmed by a selective catheterization of the GDA stump. The first episode in our patient could be considered a sentinel bleed [10]. Some institutions recommend an aggressive approach to sentinel bleeds; however, their prognostic value and the role of prophylactic treatment remain unclear [11].

According to the recommendations of the ISGPS, the patient was treated endovascularly. Endovascular treatment has a high success rate in the treatment of late PPH and is superior to both endoscopic measures and relaparotomy [9].

## Conclusion

Our case vividly presents the challenges of postoperative care after advanced pancreatic surgery. An in-depth understanding of the altered anatomy and possible complications, as well as multidisciplinary collaborations and modern facilities, should be the cornerstones of contemporary treatment of pancreatic surgical complications.

## Data Availability

Not applicable.

## References

[1] Yekebas EF, Wolfram L, Cataldegirmen G, et al. Postpancreatectomy hemorrhage: diagnosis and treatment an analysis in 1669 consecutive pancreatic resections. Ann Surg. 2007. 10.1097/01.sla.0000262953.77735.db. PMC1933568 17667506

[2] Gooiker GA, Lemmens VEPP, Besselink MG, et al. Impact of centralization of pancreatic cancer surgery on resection rates and survival. Br J Surg. 2014;101(8):1000–5. 10.1002/bjs.9468. 24844590

[3] Ho CK, Kleeff J, Friess H, Büchler MW. Complications of pancreatic surgery. HPB. 2005;7(2):99–108. 10.1080/13651820510028936. PMC2023932 18333171

[4] Williamsson C, Karlsson N, Sturesson C, Lindell G, Andersson R, Tingstedt B. Impact of a fast-track surgery programme for pancreaticoduodenectomy. Br J Surg. 2015;102(9):1133–41. 10.1002/bjs.9856. 26042725

[5] Wente MN, Veit JA, Bassi C, et al. Postpancreatectomy hemorrhage (PPH)—an International Study Group of Pancreatic Surgery (ISGPS) definition. Surgery. 2007;142(1):20–5. 10.1016/j.surg.2007.02.001. 17629996

[6] Ansari D, Tingstedt B, Lindell G, Keussen I, Ansari D, Andersson R. Hemorrhage after major pancreatic resection: incidence, risk factors, management, and outcome. Scand J Surg. 2017;106(1):47–53. 10.1177/1457496916631854. 26929287

[7] Brodie B, Kocher HM. Systematic review of the incidence, presentation and management of gastroduodenal artery pseudoaneurysm after pancreatic resection. BJS Open. 2019;3(6):735–42. 10.1002/bjs5.50210. PMC6887902 31832579

[8] Feng F, Cao X, Liu X, et al. Two forms of one complication: late erosive and nonerosive postpancreatectomy hemorrhage following laparoscopic pancreaticoduodenectomy. Medicine (Baltimore). 2019;98(30):e16394. 10.1097/MD.0000000000016394. PMC6709069 31348239

[9] Floortje Van Oosten A, Smits FJ, El D, Van Den Heuvel AF, Van Santvoort HC. Quintus Molenaar I. Diagnosis and management of postpancreatectomy hemorrhage: a systematic review and meta-analysis. HPB. 2019. 10.1016/j.hpb.2019.02.011. 30962134

[10] Brodsky JT, Turnbull ADM. Arterial hemorrhage after pancreatoduodenectomy: the ‘sentinel bleed.’ Arch Surg. 1991;126(8):1037–40. 10.1001/archsurg.1991.01410320127019. 1863209

[11] Tien YW, Wu YM, Liu KL, Ho CM, Lee PH. Angiography is indicated for every sentinel bleed after pancreaticoduodenectomy. Ann Surg Oncol. 2008;15(7):1855–61. 10.1245/s10434-008-9894-1. 18415651

